# The ABCs of the 2023 AHA/ACC/ACCP/ASPC/NLA/PCNA guideline for the management of patients with chronic coronary disease

**DOI:** 10.1002/clc.24284

**Published:** 2024-05-20

**Authors:** Michael Khorsandi, Roger S. Blumenthal, Michael J. Blaha, Payal Kohli

**Affiliations:** ^1^ Johns Hopkins Ciccarone Center for the Prevention of Cardiovascular Disease Baltimore Maryland USA; ^2^ Cardiology Division, Department of Medicine Duke University Durham North Carolina USA

**Keywords:** cardiovascular disease, chronic coronary disease, cholesterol, diabetes mellitus

## Abstract

**Background:**

The 2023 Multisociety Guideline for the Management of Chronic Coronary Disease (CCD) updates recommendations for CCD, formerly known as “stable ischemic heart disease.” This condition encompasses a spectrum of coronary vascular pathologies from subclinical to clinical ischemic heart disease.

**Hypothesis:**

The new “ABC” mnemonic offers clinicians a streamlined framework for applying Class One Recommendations (COR1) and integrating recent updates into CCD management.

**Methods:**

A critical analysis of the 2023 CCD guidelines was conducted, with this review highlighting key elements.

**Results:**

The review outlines crucial changes, including novel recommendations supported by current clinical evidence. The focus is on these developments, clarifying their importance for day‐to‐day clinical practice.

**Conclusions:**

The review encourages a synergistic approach between primary healthcare providers and cardiologists to develop comprehensive strategies for lifestyle modification and medication therapy in CCD care. Furthermore, it suggests that utilizing comprehensive risk assessment tools can refine medical decision‐making, ultimately enhancing patient care and clinical outcomes.

## Key points

- Evaluate the need for ongoing beta‐blocker therapy more than 1 year following myocardial infarction based on comorbidities (Class of recommendation 2b(COR2b)).
- Sodium‐glucose cotransporter 2 inhibitors and glucagon‐like peptide‐1 receptor agonists are recommended for selected chronic coronary disease patients, even in those without diabetes (COR1).
- Low‐dose colchicine (0.5 mg once daily) can be considered for risk reduction in secondary prevention patients with residual inflammatory risk, based on the Low Dose Colchicine 2 (LoDoCo2) trial findings (COR2b).

## Graphical abstract

A focused ABCs guide to the key elements of the 2023 Multisociety Guideline for the Management of Chronic Coronary Disease, including the class I and the newly incorporated class II recommendations.

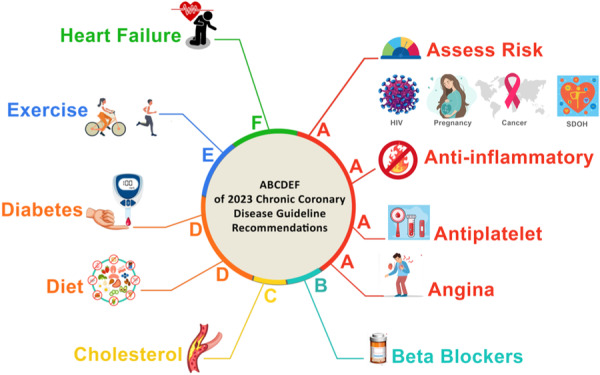

## 1 INTRODUCTION

**Figure 1:**
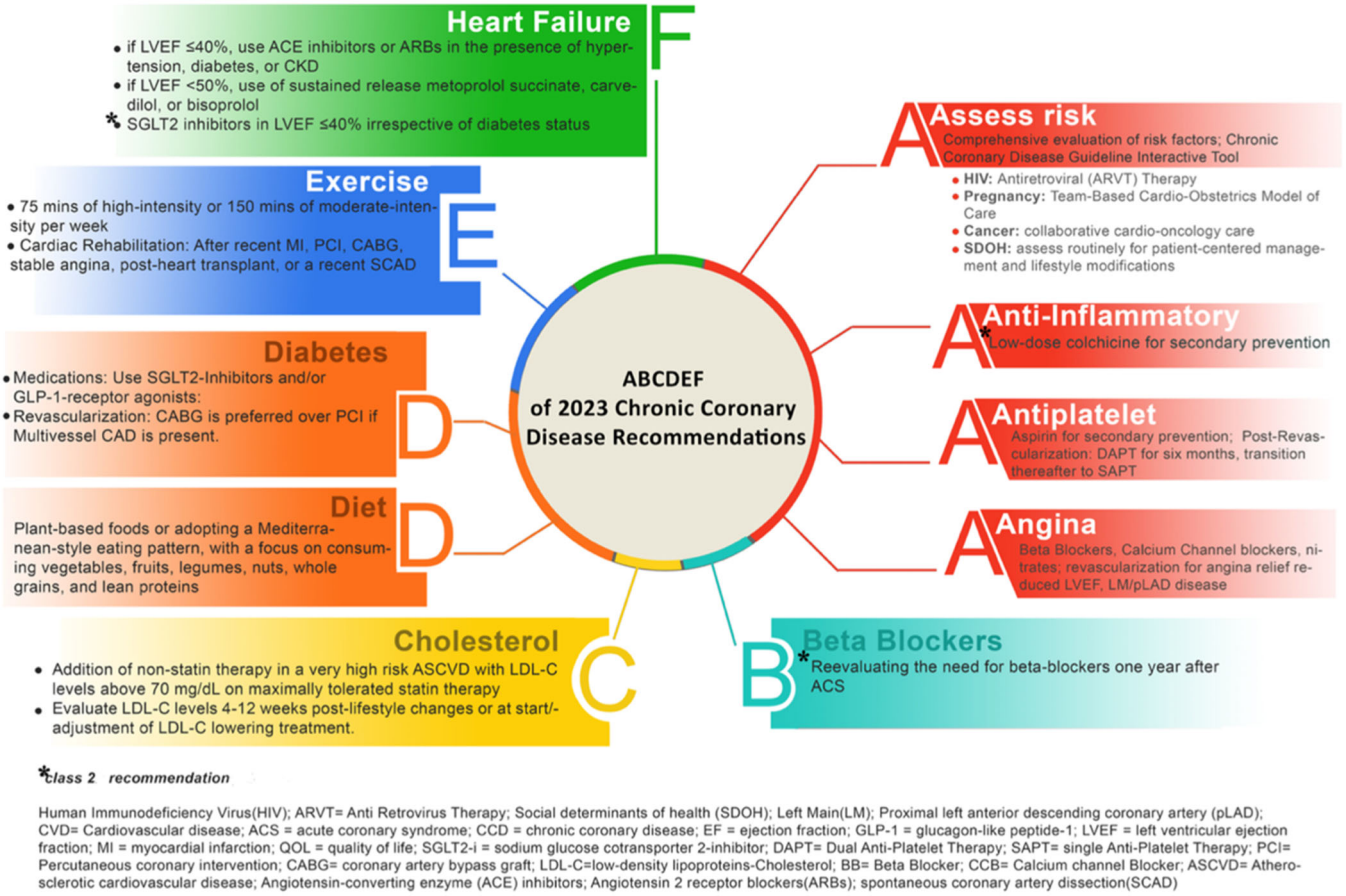
ABCDEF of the class I and the newly incorporated class II recommendations of the 2023 Guideline for Chronic Coronary Disease Management.

The 2023 AHA/ACC/Multisociety Guideline for Chronic Coronary Disease (CCD) provides updated recommendations for the management of chronic coronary disease, advocating a much more patient‐centered approach incorporating social determinants of health (SDOH). This guideline aims to extend patient survival and enhance quality of life (QoL). This review synthesizes the core perspectives from the 2023 Multi‐society guideline on CCD management, highlighting the top take‐home messages. By adopting the ABC mnemonic, it provides busy clinicians with a succinct, focused guide to the key elements of the guidelines, ensuring that the Class 1 recommendations (COR1), and newly incorporated recommendations are accessible (Figure 1).

### 1.1 Assess risk

CCD management begins with a thorough risk assessment, identifying both traditional and nontraditional risk factors. Historically, the 2012 and 2014 ACC/AHA guidelines for managing Stable Ischemic Heart Disease recommended stratifying CCD patients into categories based on the annual likelihood of major adverse cardiovascular events (MACE): low (<1%), intermediate (1%–3%), and high (>3%).^1^, ^2^ Initial risk stratification models, which lacked incorporation of clinical nuances and functional and anatomical testing data, lacked precision.^3^ However, subsequent analyses from randomized trials demonstrated that including a broader range of clinical variables and imaging data can refine risk predictions.^4^

Noninvasive test results alone might not suffice for accurate annual risk stratification in terms of cardiovascular mortality or nonfatal myocardial infarction (MI).^5^ Conversely, prior randomized trials indicated that routine revascularization did not necessarily decrease MACE rates.^6^ Nonetheless, a comprehensive risk assessment helps identify patient subsets, such as those with persistent angina, reduced left ventricular (LV) function, or heart failure (HF), who might benefit from invasive interventions.^6^

The latest 2023 CCD Guideline advocates for nuanced patient risk stratification using both noninvasive and invasive diagnostic tests, alongside validated risk scores, maintaining the categorization into low, intermediate, and high annual MACE risk.^7^ It emphasizes the importance of integrating patient demographics, medical and mental health histories, and SDOH to more accurately estimate risk.^7^

The CCD Guideline Interactive Tool aids clinicians at the bedside. It uses nine questions to generate new recommendations and prompt medication re‐evaluation at each patient visit, with a focus on customizing guideline recommendations to the individual characteristics of each patient.^7^

Clinicians should also assess patients for nontraditional risk factors that often stem from complex conditions or circumstances that require comprehensive, patient‐centered care, such as autoimmune diseases, human immunodeficiency virus (HIV), adverse pregnancy outcomes, and cancer.

#### 1.1.1 HIV

HIV and other chronic inflammatory conditions are associated with accelerated atherosclerosis and premature cardiovascular disease (CVD). Higher CD4 cell count and lower HIV viral load are associated with a lower risk of ASCVD.^8^ In adults with CCD and HIV, antiretroviral therapy (ARVT) decreases the risk of cardiovascular events.^7^, ^9^

**Table:** 

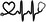 **Clinical Pearl:** CCD care for HIV‐positive patients
○ Compared with uninterrupted use of antiretroviral therapy, intermittent use increases the risk of opportunistic disease and death from any cause, including CVD.^10^ ○ There is increased cardiovascular risk in individuals with HIV, which is likely related to the adverse impact of antiretroviral treatments on lipid profiles.^11^ ○ It is recommended to choose newer generation antiretroviral treatment regimens associated with more favorable lipid and cardiovascular risk profiles, and less interaction with statins and other CCD medications.^12^ ○ Individuals with HIV and CVD should choose an alternative instead of an abacavir‐containing regimen because of its possible association with increased cardiovascular events.^13^

#### 1.1.2 Adverse pregnancy outcomes

The CCD guidelines recognize the team‐based Cardio‐Obstetrics model of care as a multidisciplinary, collaborative approach where specialists work in concert with the patient, to address health concerns. This approach includes risk stratification and counseling on potential maternal, obstetric, and fetal outcomes and continues throughout the pregnancy, delivery, and the postpartum periods.^7^

#### 1.1.3 Cancer

As the prevalence of concurrent CCD and cancer increases due to an aging population with improved cancer survival rates, the field of Cardio‐Oncology has evolved. To enhance long‐term cardiovascular outcomes, guidelines advocate for a collaborative approach that combines the expertise of both cardiologists and oncologists.^7^

#### 1.1.4 Social determinant of health (SDOH)

Acknowledging that SDOH significantly influences patient outcomes in CCD and in healthcare utilization, the guideline emphasizes regular SDOH assessments. These encompass mental health, psychosocial stressors, health literacy, and sociocultural factors. Understanding SDOH helps clinicians provide equitable, culturally sensitive care and develop comprehensive plans that include community resources for nutrition, exercise, and social support.^14^ Thus, integrating SDOH assessments is crucial for tailored, patient‐centered CCD management and lifestyle modifications.^7^

### 1.2 Anti‐Inflammatory

Inflammation plays a central role in the pathogenesis of atherosclerosis, Therefore, in addition to addressing “cholesterol” risk and co‐morbid risk factors (i.e., hypertension, diabetes, obesity, sleep apnea) managing the residual “inflammatory risk” in those with well‐controlled low‐density lipoprotein cholesterol (LDL‐C) on statin therapy has become another key target in reducing MACE. This residual “inflammatory risk” can be mitigated through healthy dietary choices, regular exercise, smoking cessation, improved sleep hygiene, and stress management.^15^ However, in selected patients, pharmacological anti‐inflammatory approaches can complement background lipid‐lowering therapy to further reduce MACE.

Clinical trials have noted the beneficial effects of colchicine 0.5 mg in reducing the risk of cardiovascular events. The Colchicine Cardiovascular Outcomes Trial (COLCOT) trial showed that colchicine therapy in patients with a recent acute coronary syndrome (ACS) led to a 23% risk reduction in MACE.^16^ In a stable CCD population, the Low Dose Colchicine 2 (LoDoCo2) trial showed that colchicine improved CV outcomes (primarily MI and ischemia‐driven revascularization) among patients with CCD compared with placebo. However, there was a signal toward higher non‐CV mortality with colchicine. The etiology of this risk was unclear, but hospitalizations for infections and pneumonia were similar between the two arms.^17^ Low‐dose colchicine is now included in the CCD guidelines for secondary prevention (COR2b)^7^ to lower recurrent atherosclerotic cardiovascular disease (ASCVD) among patients with clinically stable ASCVD.

### 1.3 Antiplatelet therapy

The choice of appropriate antiplatelet therapy for CCD and post‐revascularization should be personalized for each patient, carefully weighing the risks of bleeding against the potential for thrombotic complications, while acknowledging the conundrum that the risk factors associated with increased bleeding often overlap with those indicating elevated thrombotic risk. While bleeding risk usually increases gradually over time, thrombotic risk can lead to abrupt and serious complications, particularly after recent percutaneous coronary intervention (PCI) or ACS. The 2023 CCD Guideline provides recommendations for the application of antiplatelet therapy in individuals with CCD as an update to the previous 2016 AHA/ACC guideline Focused on dual antiplatelet therapy (DAPT) and 2021 AHA/ACC guidelines for Coronary Artery Re vascularization.^18^, ^19^

#### 1.3.1 Chronic coronary disease

The effectiveness of aspirin use in lowering the occurrence of MACE for secondary prevention in ASCVD is clear.^20^, ^21^ The 2023 CCD Guideline recommends that for individuals with CCD who are not prescribed oral anticoagulants (OAC) for another indication, single antiplatelet therapy (SAPT) is usually recommended with low‐dose aspirin (75–100 mg) for reducing the incidence of atherosclerotic cardiovascular events.^7^ In addition, DAPT, combining clopidogrel with low‐dose aspirin in patients with recent ACS is recommended up to 12 months after the ACS regardless of receiving PCI or not.^7^ If aspirin is not well‐tolerated, clopidogrel is a reasonable alternative. There are contemporary trials that advocate for clopidogrel monotherapy after a short course of DAPT after PCI.^22^

The Cardiovascular Outcomes for People Using Anticoagulation Strategies (COMPASS) trial investigated the use of low‐dose rivaroxaban, with or without aspirin, in reducing ASCVD events and demonstrated increased efficacy of combining rivaroxaban with aspirin over aspirin alone.^23^ Notably, those with multivessel coronary artery disease (CAD) and at least one of other conditions like peripheral arterial disease and chronic kidney disease observed the most substantial absolute risk reduction from adding low‐dose rivaroxaban to their aspirin regimen.^24^ The 2023 CCD Guideline recommends that for those at elevated risk for recurrent ischemic episodes who do not have an indication for direct oral anticoagulant (DOAC) or DAPT, and a low‐to‐intermediate risk of bleeding, the incorporation of low‐dose rivaroxaban (2.5 mg twice daily) with low‐dose aspirin may be beneficial to address residual “thrombotic risk” (COR2a).^7^

#### 1.3.2 Post revascularization

Multiple trials have studied the optimal duration for antiplatelet therapy after revascularization. A meta‐analysis of randomized controlled trials with a cohort of 31 666 patients indicated that a shorter DAPT duration could be associated with a reduction in all‐cause mortality. Patients who received DAPT for 6 months or less exhibited mortality, MI, and stent thrombosis rates comparable to those treated for 1 year, yet they experienced less significant bleeding.^25^

The 2023 CCD Guidelines provide recommendations for patients undergoing PCI with and without the need for OAC therapy for another indication. In individuals undergoing PCI for CCD who do not require OAC, DAPT is recommended for at least 6 months postprocedure, transitioning thereafter to an SAPT.^7^ Conversely, for those who need OAC therapy post‐PCI, a 1–4‐week course of DAPT plus DOAC is suggested, followed by a 6‐month period of clopidogrel with DOAC to balance the prevention of thrombotic events with the minimization of bleeding risks.^7^

A shorter DAPT period of 1–3 months followed by P2Y12 inhibitor monotherapy extending to a year, may be appropriate for patients with a lower risk of thrombotic events (COR2a). This is supported by the ULTIMATE‐DAPT trial, a placebo‐controlled, randomized trial involving 3400 patients undergoing PCI for ACS, which showed that compared with standard 12‐month DAPT comprising aspirin and ticagrelor, 1‐month DAPT followed by de‐escalation to ticagrelor monotherapy reduces clinically relevant bleeding without increasing thrombotic risk at 1 year. Bleeding Academic Research Consortium (BARC) levels 2, 3, or 5 at 1 year occurred in 4.6% of patients continuing DAPT and 2.1% of patients on ticagrelor monotherapy (hazard ratio [HR], 0.45; 95% confidence interval [CI]: 0.30–0.66; *p* < .0001).^26^

Conversely, for selected patients with a lower risk of bleeding and a history of MI, prolonging DAPT for up to 3 years beyond the initial year may be beneficial in lowering the recurrence of MACE (COR2b).^27^ Notably, in the prevention of cardiovascular events in patients with prior heart attack Using Ticagrelor Compared to Placebo on a Background of Aspirin–Thrombolysis in Myocardial Infarction 54 (PEGASUS‐TIMI 54) trial, the addition of ticagrelor (ticagrelor 60 mg BID) to low‐dose aspirin in patients 1–3 years after MI, significantly reduced the risk of cardiovascular death, MI, or stroke.^28^

### 1.4 Angina

In the management of patients with CCD and stable angina, three primary goals should be at the forefront of any therapeutic strategy. These are the relief of angina symptoms, the prevention of nonfatal occurrences like MI, and improving long‐term survival rates.^7^

The contemporary strategies for managing CCD include revascularization, pharmacological treatment, or a synergistic approach that integrates both modalities. A systematic review has identified key survival predictors in CCD, including the anatomical and functional severity of the condition, LV function, and comorbidities such as diabetes and renal impairment.^29^ Management strategies should be customized to each patient's unique clinical profile. Some individuals may benefit from an integrated approach that combines multiple therapeutic interventions, while for others, a single treatment modality may be more advantageous. For example, in patients with CCD with severe LV dysfunction (LVEF ≤ 35%) or significant left main stenosis, coronary artery bypass grafting (CABG) has been shown to offer a survival advantage compared with medical therapy alone.^7^, ^30^, ^31^, ^32^

Revascularization approaches, including PCI or CABG, as well as pharmacologic approaches reduce symptoms of angina‐related CCD, although medical optimization is always recommended as the first‐line intervention before procedural interventions. This was previously emphasized by the results of three major RCTs: COURAGE (Clinical Outcomes Utilizing Revascularization and Aggressive Drug Evaluation), ISCHEMIA (The International Study of Comparative Effectiveness with Medical and Invasive Approaches), and BARI‐2D (Bypass Angioplasty Revascularization Investigation 2 Diabetes), which demonstrated lack of reduction in MACE with nonurgent cardiovascular revascularization beyond the benefit of optimal pharmacologic Goal Directed Medical Therapy (GDMT).^33^, ^34^, ^35^, ^36^ Clinicians should prioritize the optimization of GDMT to reduce MACE. It is important to emphasize that in the presence of Left Main disease, or reduced left ventricular ejection fraction (LVEF)/heart failure with reduced ejection fraction (HFrEF),^34^ revascularization is often recommended.

#### 1.4.1 Pharmacologic approach

For the management of angina, the initial treatment strategy involves the use of antianginal medications, such as beta blockers, calcium channel blockers (CCB), or long‐acting nitrates, to alleviate symptoms. If symptoms persist, incorporating an additional antianginal agent from a distinct class, such as ranolazine, is recommended.^7^

#### 1.4.2 Revascularization approach

While historical studies implied that surgical revascularization could improve survival rates for patients with CCD,^37^, ^38^ recent studies and comprehensive reviews have largely found no substantial impact of such procedures on reducing all‐cause mortality in CCD patients. A meta‐analysis of randomized trials showed an annualized mortality rate in the medical therapy arm, ACME‐2 (Angioplasty Compared to Medicine: Two‐Vessel Disease) has reported 4.0%, while 1.0% in medical therapy arm of later studies like FAME‐2 (Fractional Flow Reserve Guided Percutaneous Coronary Intervention Plus Optimal Medical Treatment [OMT] vs. OMT), signifying progress in medical management.^29^

The 2023 CCD Guideline, similar to the previous 2021 Guideline for Coronary Artery Revascularization, emphasizes the importance of informed decisions regarding revascularization.^7^, ^19^ There should be a comprehensive, team‐based approach, the guidelines advocate for the involvement of a Heart Team, including interventional cardiologists, cardiac surgeons, and other cardiovascular specialists to improve patient outcomes.^7^ This team plays a fundamental role in devising revascularization strategies that are tailored to individual patient profiles, especially those with complex coronary or multivessel diseases and other significant comorbidities.

The principle of patient‐centered care is also emphasized. Treatment decisions should reflect the best clinical evidence and align with patients’ values, preferences, and goals, reinforcing the practice of shared decision‐making. The 2023 CCD Guideline recommends that for patients with persistent angina that limits their lifestyle despite optimal medical therapy with a disease burden that is amenable to revascularization, revascularization should be an attempt to alleviate symptoms.^7^

### 1.5 Beta blockers

Prior guidelines have advocated for extended duration of beta‐blocker therapy, especially post‐MI or ACS and continued for 3 years and in CCD. However, a major shift in the guidelines now recommends re‐evaluating the indication for beta‐blockers 1 year after a myocardial event, given the mixed evidence on their long‐term efficacy, possibility of side effects like fatigue and depression, along with possible drug interactions (COR2b).^7^ Ongoing beta‐blocker therapy is now specifically indicated only for patients with reduced left ventricular ejection fraction (LVEF < 50%), presenting with angina, arrhythmias, hypertension, or who have experienced spontaneous coronary artery dissection.^7^ Beta‐blockers are generally not recommended for CCD patients receiving elective PCI, those without prior MI, or those with LVEF above 50%.^7^, ^39^

### 1.6 Cholesterol/Lipid management

Aggressive lowering of LDL‐C remains a pillar of CCD management. The 2018 AHA/ACC/Multisociety Guideline emphasized an LDL‐C threshold of ≤70 mg/dL or lower in individuals at high risk of ASCVD and to consider the use of non‐statin LDL‐C lowering medications.^40^ The 2019 ESC/EAS Dyslipidemia Guideline advocated for an even lower threshold LDL‐C of <55 mg/dL or an LDL‐C lowering >50%, whichever is lower.^41^ In 2022, the American College of Cardiology (ACC) Expert Consensus Decision Pathway (ECDP) on the role of non‐statin therapies for LDL‐C lowering in the management of ASCVD risk discussed three Food and Drug Administration (FDA)‐approved non‐statin therapies (bempedoic acid, evinacumab, inclisiran, and proprotein convertase subtilisin/kexin type 9 (PCSK9) monoclonal antibodies), which were not previously recommended in the 2018 American Heart Association (AHA)/ACC cholesterol guideline.^42^

The ECDP also recommends that for patients at very high risk of ASCVD, or those with ASCVD confirmed to have familial hypercholesterolemia (FH), adding nonstatin therapies if their LDL‐C levels are 55 mg/dL or higher, on a background of high‐intensity statin treatments is recommended. For those with ASCVD who are not considered at very high risk or who have LDL‐C levels over 190 mg/dL without confirmed FH, a higher threshold—over 70 mg/dL for LDL—triggers the addition of nonstatin therapies alongside the maximum tolerated statin treatments. Ezetimibe with or without PCSK9 inhibitor monoclonal antibodies is the recommended first choice for a nonstatin agent if the desired LDL‐C reduction is <25%; otherwise a monoclonal antibody PCSK9 inhibitor may be preferred.^42^

Evaluation of LDL‐C between 4 and 12 weeks following dose titration is recommended to rapidly adjust dose as needed.^7^ Regular lipid assessment promotes adherence to the prescribed regimen, identifies patients who may need more aggressive treatment, and mitigates the risks of treatment delays. Patients failing to reach targeted LDL‐C levels require combination lipid‐lowering strategies up front or early. The Further Cardiovascular Outcomes Research with Proprotein convertase subtilisin/kexin type 9 serine protease (PCSK9) Inhibition in Subjects with Elevated Risk (FOURIER) study illustrated that adding evolocumab to statins decreased cardiovascular events and significantly reduced LDL‐C levels,^43^ with The FOURIER Open‐Label Extension (FOURIER‐OLE) study confirming the sustained safety and efficacy of PCSK9 inhibitors.^44^ Furthermore, the Evaluation of Cardiovascular Outcomes After an Acute Coronary Syndrome During Treatment with Alirocumab (ODYSSEY OUTCOMES) trial evaluated alirocumab use in patients with a recent 1–12 months history of ACS, who were already on maximum doses of statins, with or without ezetimibe. The trial reported a 15% relative risk reduction in cardiovascular events among patients treated with alirocumab, with the most notable benefits observed in those with additional high‐risk clinical factors.^45^

Evidence from the FOURIER and Evaluating PCSK9 Binding Antibody Influence on Cognitive Health in High Cardiovascular Risk Subjects (EBBINGHAUS) trials confirmed the safety of the very low LDL‐C (medial LDL‐C in FOURIER of 26 mg/dL), with no additional neurocognitive events in those with very low LDL‐C.^46^, ^47^

In the Reduction of Cardiovascular Events with Icosapent Ethyl−Intervention (REDUCE‐IT) trial, icosapent ethyl, a purified derivative of eicosapentaenoic acid significantly reduced the relative risk of MACE by 25% and cardiovascular death by 20% in individuals with well‐controlled LDL‐C with established ASCVD or additional risk factors and TG 150‐499 mg/dL.^48^ On the other hand, the Study to Assess Statin Residual Risk with Epanova in High Cardiovascular Risk Patients with Hypertriglyceridemia (STRENGTH) trial found no statistically significant benefit from a mixed omega‐3 fatty acid formulation compared with a placebo.^49^ Variations in outcomes between these trials may be attributed to differences in eicosapentaenoic acid (EPA) dosage, bioavailability, and serum concentration of on‐treatment EPA. It is unclear if the use of mineral oil as the comparator in the REDUCE‐IT trial magnified the apparent benefit of high dose EPA since mineral oil increased LDL‐C levels by 11% and impacted inflammatory markers, although the magnitude of the benefit of icosapent ethyl likely outweighs the relative risk increase conferred by elevation in LDL‐C.

### 1.7 Diet

Prior clinical studies in secondary prevention cohorts have demonstrated a reduced risk of ASCVD events and all‐cause mortality among individuals adhering to plant‐based, Mediterranean, or low‐sodium dietary patterns.^50^, ^51^ In the Long‐term secondary prevention of cardiovascular disease with a Mediterranean diet and a low‐fat diet (CORDIOPREV) randomized trial which included patients with established coronary heart disease, a Mediterranean diet was superior to a low‐fat diet in preventing MACE (Mediterranean diet crude rate 28/1000 person‐years [95% CI: 27.9–28.3] vs. low‐fat group 37.7/1000 person‐years [95% CI: 37.5–37.9], log‐rank *p* = .039).^52^ In addition, limiting sodium to <2300 mg/day lowers blood pressure and cardiovascular risk.

For reducing cardiovascular disease events, the 2023 CCD Guideline recommends following a diet focused on plant‐based foods and adopting a Mediterranean‐style eating pattern, with a focus on consuming more vegetables, fruits, legumes, nuts, whole grains, and lean proteins.^7^ In addition, those with CCD refrain from using nonprescription dietary supplements such as fish oil, omega‐3 fatty acids, or vitamins, as there is insufficient evidence to suggest these supplements have a beneficial impact on cardiovascular event reduction.

### 1.8 Diabetes

#### 1.8.1 Medications

To significantly decrease cardiovascular risk in individuals with type 2 diabetes as ASCVD, a coordinated approach is crucial, incorporating lifestyle adjustments, nutritional counseling, and outcomes‐based medications.^53^ Meta‐analysis of six randomized controlled trials, demonstrated that sodium‐glucose cotransporter‐2 (SGLT2) inhibitors substantially reduce MACE, particularly decreasing the risk of HF hospitalization and progression of kidney disease in type 2 diabetes patients.^54^ Similarly, an analysis of eight trials revealed that glucagon‐like peptide‐1 receptor agonists (GLP‐1RAs) not only lower MACE but also contribute significantly to reducing all‐cause mortality, HF hospitalizations, and the progression of kidney disease.^55^

The Semaglutide and Cardiovascular Outcomes in Obesity without Diabetes (SELECT) study, a randomized controlled trial for individuals over 45 years with pre‐existing cardiovascular disease and a higher body mass index but without diabetes, evaluated the impact of semaglutide on cardiovascular events.^56^ Over an average span of 33 months, semaglutide users, in comparison to those on placebo, exhibited a 20% reduction in the composite outcome of death from cardiovascular causes, nonfatal MI, or nonfatal stroke. There was a consistent trend toward reduction in each component of the composite outcome, as well as in all‐cause mortality, which also declined by approximately 20%. In addition, relative to placebo, patients using semaglutide saw decreases in hospitalizations due to HF, particularly those with higher baseline hemoglobin A1C levels, along with significant improvements in systolic blood pressure and body weight.

#### 1.8.2 Revascularization

The presence of diabetes mellitus in CCD significantly impacts the outcomes and decisions surrounding coronary revascularization. Several landmark studies have delved into the complex interplay between diabetes mellitus and revascularization.^35^, ^57^, ^58^ In patients with type 2 diabetes and multivessel coronary artery disease involving the left anterior descending artery (LAD), CABG is recommended over PCI, with an emphasis on using the left internal mammary artery graft to decrease the likelihood of death and the need for subsequent revascularization procedures.^7^

### 1.9 Exercise

Patients with CCD should engage in regular physical exercise. At least 150 min of moderate‐intensity aerobic activities per week or at least 75 min of high‐intensity aerobic activities per week is recommended to enhance functional capacity and quality of life (QOL) while reducing hospital admissions and mortality rates.^7^, ^59^ In addition, patients are encouraged to engage in resistance or strength training exercises at least twice a week, which not only boosts muscle strength but also improves functional capacity, if no contraindications are present.^7^

Contraindications to exercise in patients with severe, potentially fatal, and unstable conditions include conditions such as unstable angina and other high‐risk cardiovascular disorders including advanced arrhythmias, decompensated cardiac failure, or active thromboembolism. In addition, significant noncardiovascular diseases that pose immediate life risks, such as acute infections, poorly managed diabetes mellitus, terminal malignancies, or severe mental health challenges, may also preclude regular physical exercise.

#### 1.9.1 Cardiac rehabilitation (CR)

Patients with CCD and a recent MI, PCI, CABG, stable angina, postheart transplant, or a recent spontaneous coronary artery dissection should enroll in a CR program.^7^ Those who participate in CR have significantly better outcomes compared with those who do not participate, including lower all‐cause and cardio‐vascular mortality rates, lower rehospitalization rates (total, cardiovascular, and noncardiovascular), and superior QOL.^59^, ^60^

### 1.10 HF

Primary care clinicians and cardiologists play a pivotal role in the management of guideline‐directed medical therapy for HF. In randomized controlled trials (RCTs), renin‐angiotensin‐aldosterone system inhibitors (RAASi) improve symptoms, reduce hospitalization rates, and prolong survival for high‐risk patients with CCD.^61^, ^62^ In addition, the 2022 AHA/ACC/HFSA HF guideline recommends this therapy for patients with CCD and an LVEF of 40% or less, who exhibit symptoms beyond stage B.^63^

On the other hand, key trials such as the heart outcomes prevention evaluation trial have demonstrated significant benefits of ramipril in patients with CCD and preserved LVEF, including reductions in death, cardiovascular events, and stroke.^62^ However, studies such as Quinapril Ischemic Event (QUIET) and Comparison of Amlodipine versus Enalapril to Limit Occurrence of Thrombosis (CAMELOT) suggest that RAASi's effect on atherosclerosis progression and MACE in patients with CCD, normal blood pressure, and preserved LVEF is minimal, except in those with chronic kidney disease (CKD).^64^, ^65^ This indicates that the benefits of RAASi may depend on the broader clinical context and the combined management strategies for CCD. In patients with CCD who also have hypertension, diabetes, LVEF ≤ 40%, or CKD, the use of ACE inhibitors, or ARBs for those intolerant to ACE inhibitors, is recommended.^7^

For patients with an LVEF below 50%, the 2023 CCD Guideline recommends the use of maximally tolerated sustained‐release metoprolol succinate, carvedilol, or bisoprolol.^7^ Additionally, for those with HF and LVEF of 40% or lower, the recommendation is to initiate therapy with SGLT2 inhibitors to reduce the risk of cardiovascular death and HF hospitalization and to improve QOL, regardless of whether the patient has diabetes (COR1).^7^ Conversely, in patients with CCD and LVEF > 40%, the use of an SGLT2 inhibitor can be beneficial in decreasing HF hospitalizations and to improve QOL irrespective of diabetes status (COR2a).^7^

## 2 CONCLUSION

In the 2023 AHA/ACC Guideline on CCD management, several key Class I, and new recommendations have been introduced. These guidelines facilitate a collaborative approach between primary healthcare providers and cardiologists, promoting the development of comprehensive strategies in both lifestyle modification and medication therapy for individuals with CCD. It endorses the use of advanced lipid‐lowering therapies such as PCSK9 inhibitors, sodium‐glucose cotransporter‐2 inhibitors, glucagon‐like peptide‐1 receptor agonists, and low‐dose colchicine for selected patients with CCD. With the “ABC” framework, the guidelines efficiently highlight key areas where primary healthcare providers and internists can improve the implementation of outcomes‐based medications in CCD.

Additionally, the implementation of comprehensive risk assessment tools, like the CCD Guideline Interactive Tool^7^ can enhance medical decision‐making. These tools should be rigorously validated to accurately predict MACE in contemporary CCD cohorts. They must account for a wide range of factors, including patient demographics, medical history, social determinants, and results from both noninvasive and invasive diagnostic tests.

## AUTHOR CONTRIBUTIONS

All authors had access to the data accessed in this study and made substantial contributions in the creation of this manuscript.

## CONFLICT OF INTEREST STATEMENT

The authors declare no conflict of interest.

## Data Availability

Data sharing is not applicable to this article as no new data were created or analyzed in this study.
